# Case Report: Ribociclib-induced phototoxicity presented as dyschromia with subsequent bullae formation

**DOI:** 10.3389/fonc.2023.1184738

**Published:** 2023-08-24

**Authors:** Jyun-Yan Jhan, Wei-En Wang, Sung-Chao Chu, Chiu-Hsuan Cheng, Chung-Hsing Chang

**Affiliations:** ^1^ Skin Institute, Department of Dermatology, Hualien Tzu Chi Hospital, Buddhist Tzu Chi Medical Foundation, Hualien, Taiwan; ^2^ Department of Hematology-Oncology, Buddhist Tzu Chi General Hospital, Hualien, Taiwan; ^3^ Department of Pathology, Buddhist Tzu Chi General Hospital, Hualien, Taiwan; ^4^ Doctoral Degree Program in Translational Medicine, Tzu Chi University and Academia Sinica, Taipei, Taiwan; ^5^ Institute of Medical Sciences, College of Medicine, Tzu Chi University, Hualien, Taiwan

**Keywords:** breast cancer, cyclin-dependent kinases 4/6 inhibitor, ribociclib, drug allergy, dyschromia, phototoxicity, bullae, case report

## Abstract

Ribociclib, a cyclin-dependent kinase 4/6 inhibitor, is a novel targeted therapy for advanced-stage breast cancer. Although ribociclib-induced cutaneous side effects have been previously noted, they have not been well documented. Herein, we present a case of ribociclib-induced phototoxicity, which manifested as dyschromia over sun-exposed forearms and neck initially and as bullae formation subsequently. A 71-year-old woman with metastatic breast cancer developed dyschromia after daily treatment with ribociclib (600 mg) for 7 months. Skin biopsy of the pigmented lesion revealed interface dermatitis with melanin incontinence and dyskeratotic cells and ballooning keratinocytes with loss of melanocytes in the basal layer. Further, clefting at the basal layer of epidermis was noted in a more hyperpigmented field. Fontana–Masson staining revealed melanophages in the dermis. Human Melanoma Black-45 staining revealed decreased melanocyte numbers in the epidermis above the cleft. Immunohistochemical analyses revealed activated CD1a+ epidermal Langerhans cells and infiltrating CD4+ and CD8+ T cells in the epidermis and dermis, thereby indicating type IV hypersensitivity that was associated with damage to keratinocytes and melanocytes. To prevent progression of bullous dermatitis, we advised the patient to discontinue ribociclib and prescribed oral and topical prednisolone. Due to the risk of phototoxicity, we educated the patient on sun-protection strategies. The patient’s skin lesions subsided during the 2 months of treatment. Phototoxicity with dyschromia is a rare but significant ribociclib-induced cutaneous side effect. Early diagnosis, rapid ribociclib withdrawal, protection from sunlight, and prompt treatment are critical for preventing subsequent severe bullous dermatosis.

## 1 Introduction

Breast cancer is one of the leading causes of death among patients with malignant tumors. In 2020, breast cancer was responsible for 680,000 deaths worldwide (1). Recently, the anti-breast cancer therapeutic plan was established based on staging and hormone receptors (HRs). Previously, patients with advanced-stage breast cancer of an HR-positive (HR+) and a human epidermal growth factor receptor 2-negative (HER2−) status were usually treated with single-agent endocrine therapy; however, these treatments did not achieve long-lasting effects due to drug resistance (2, 3).

In recent years, several classes of targeted therapies have been developed for the treatment of breast cancer. These include cyclin-dependent kinase (CDK) 4/6 inhibitors, including palbociclib, ribociclib, and abemaciclib. These drug therapies have been approved by the Food and Drug Administration for the treatment of metastatic breast cancer with (HR+)/(HER2−) expression (4, 5).

CDKs are a group of kinases that regulate the cell cycle, and CDK4/6 modulates G1/S phase transition during DNA synthesis; this cell cycle control system is usually disrupted in cancers. The estrogen receptor–cyclin D1-CDK4/6-retinoblastoma pathway plays an important role in estrogen receptor-positive breast cancer development. Therefore, CDK4/6 inhibitors exert an anti-cancer effect through the inhibition of this pathway (6).

Previous studies have revealed the common adverse effects of ribociclib; these include neutropenia, anemia, fatigue, diarrhea, a prolonged QTc, and an elevated liver function (7, 8). However, the important, severe dermatological side effects of this drug have not been sufficiently documented. Herein, we present a case of ribociclib-induced phototoxicity and bullae formation.

## 2 Case presentation

A 71-year-old woman presented with hypertension, hypertriglyceridemia, and diabetes mellitus. A mass in the right breast of the patient was initially observed at least 2 years ago, and the patient had visited the general surgery outpatient department because of a fungating lesion that had persisted for 3 months. A follow-up tumor biopsy had revealed infiltrating lobular carcinoma luminal B1 (estrogen receptor: 70%, progesterone receptor: 30%; HER2–). Positron emission tomography revealed pleural seeding and, liver and bone metastasis. Hence, the patient was newly diagnosed with a right inflammatory breast-infiltrating lobular carcinoma with multiple metastases (cT4N3cM1, stage IV). Subsequently, the patient received a daily combination therapy of letrozole (an aromatase inhibitor) and ribociclib (600 mg) along with a monthly injection of denosumab.

**Figure 1:**
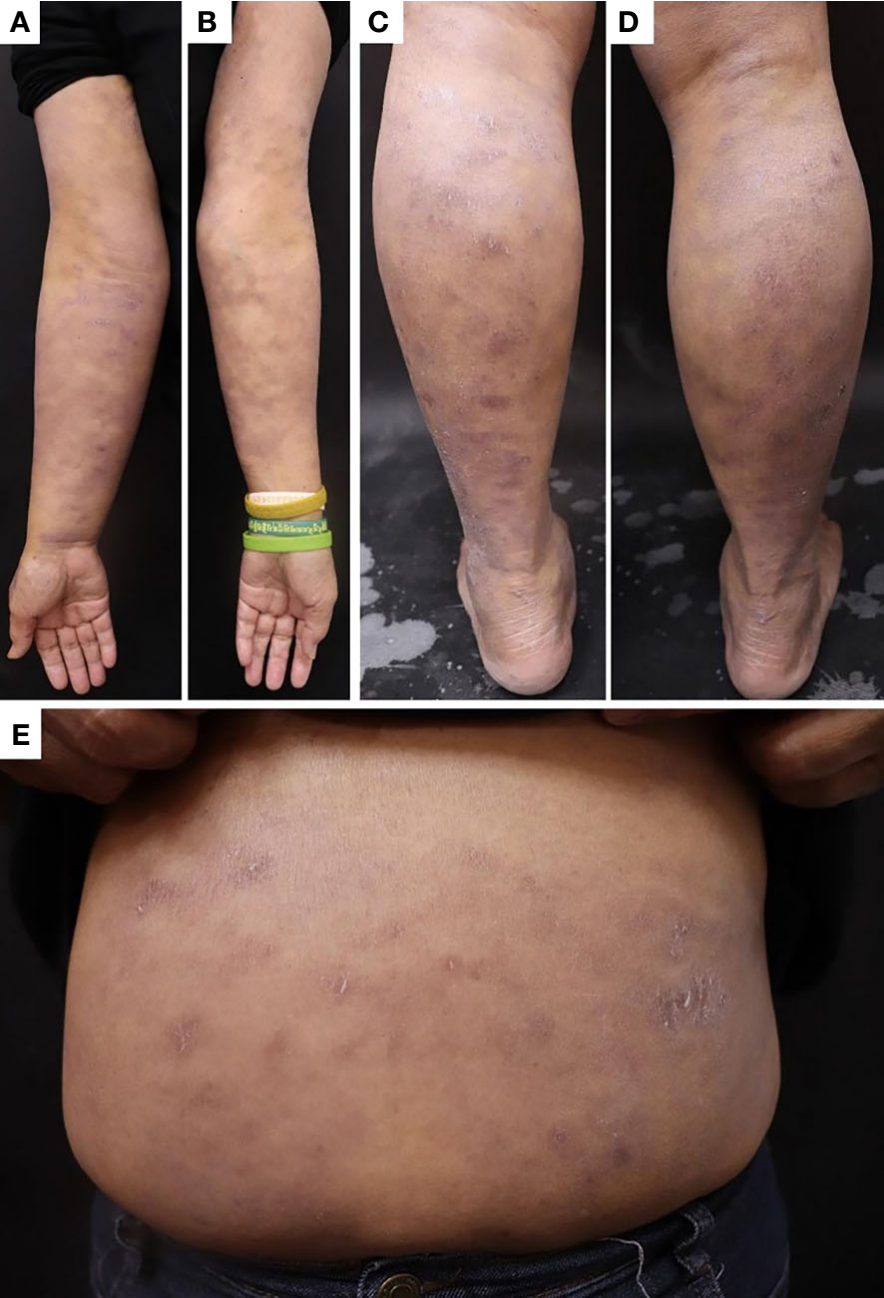
Skin pigmentation in the **(A)** right forearm, **(B)** left forearm, **(C)** left lower leg, **(D)** right lower leg, and **(E)** trunk.

**Figure 2:**
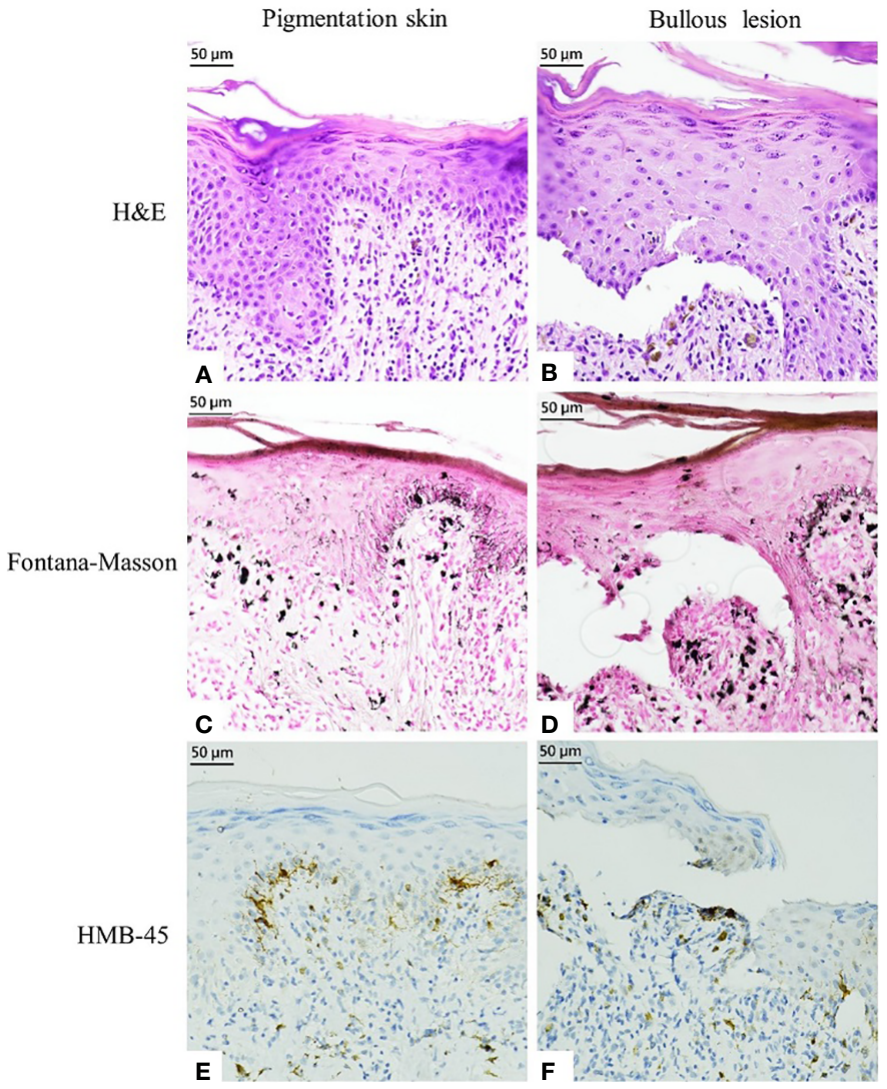
Histopathological examination and immunostaining finding of pigmented markers in the pigmented skin and bullous lesion. **(A, B)** Hematoxylin and eosin staining. **(C, D)** Fontana–Masson Staining. **(E, F)** HMB-45 staining.

Seven months later, the patient developed grayish pigmentation on the four limbs and trunk; these were especially prominent on the sunlight-exposed forearms (**Figure 1**). The patient reported progressive pigmentation and pruritus of the lesion. Laboratory data revealed no neutropenia. Thereafter, the department of hematology referred the patient to the dermatology outpatient clinic for further evaluation. A skin biopsy examination of the pigmented lesion was performed; pathological examination revealed interface dermatitis with melanin incontinence. Notably, dyskeratotic cells and clefting at the basal layer of epidermis were identified (**Figures 2A, B**). Fontana–Masson staining for melanin revealed that the melanophages was distributed unevenly and generally present in the upper dermis (**Figures 2C, D**). Human Melanoma Black-45 (HMB-45) staining revealed decreased melanocyte numbers in both normal and clefting skin lesions (**Figures 2E, F**). Therefore, the hyperpigmented skin lesion was speculated to have been caused by altered melanin distribution and melanocyte numbers.

**Figure 3:**
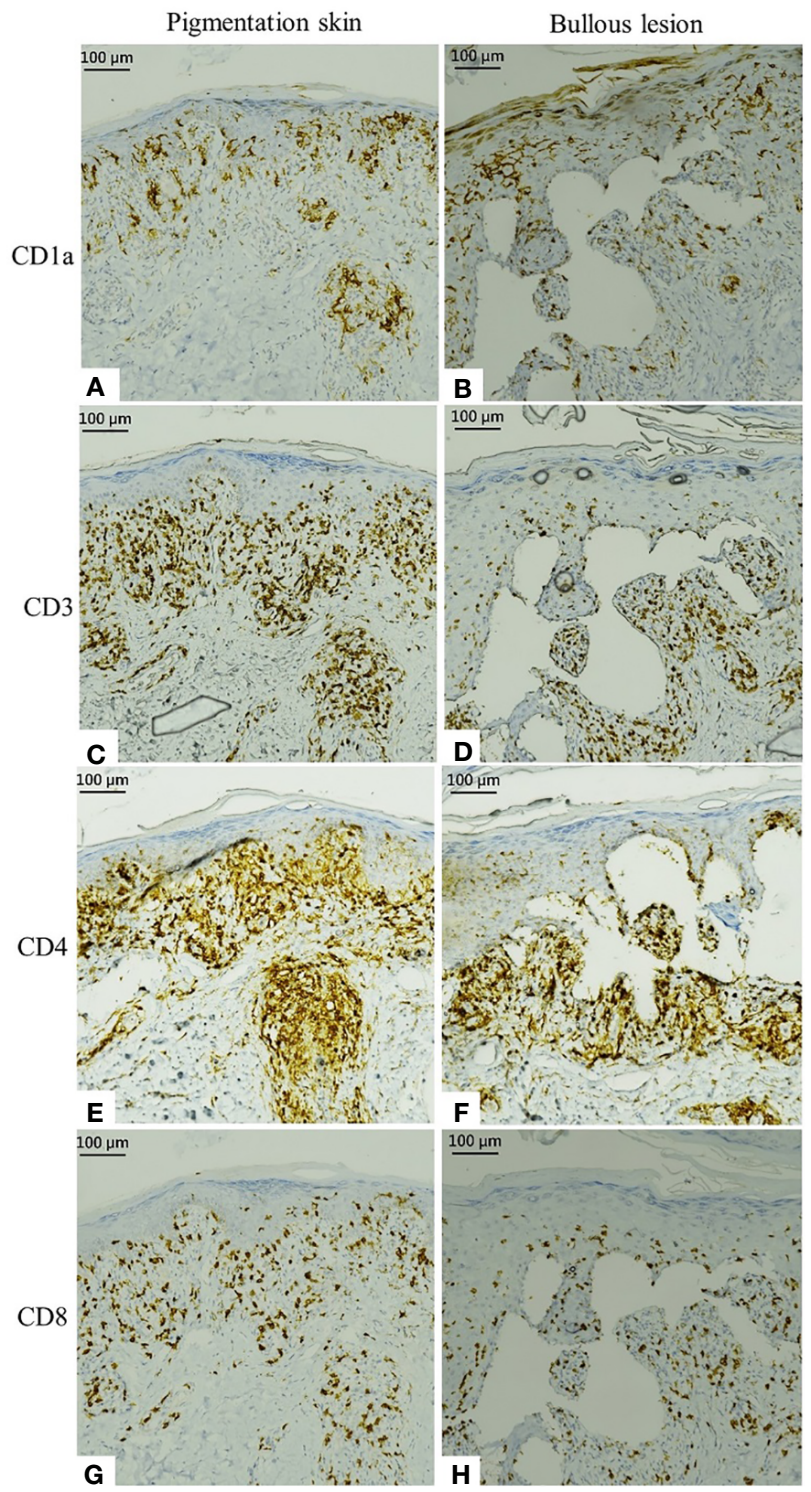
Immunostaining of specimens from the pigmented skin and bullous lesion. **(A, B)** Activated epidermal Langerhans cells present with CD1a. **(C, D)** Immunohistochemical staining of CD3. **(E, F)** Infiltrating CD4. **(G, H)** Infiltrating CD8.

Further, immunohistochemical staining revealed activated epidermal Langerhans cells presenting with CD1a as well as CD3+, CD4+, and CD8+ T cells infiltrating into the epidermis and dermis; these findings indicated type IV hypersensitivity (**Figures 3A–H**). Thus, a clinicopathological diagnosis of ribociclib-induced phototoxicity, manifesting initially as dyschromia and subsequently as bullous dermatosis, was made.

Owing to the severe dermatological side effects, we advised the hematologist to withdraw ribociclib and prescribe oral (20 mg/day) and topical prednisolone instead; however, treatment with letrozole was continued. Additionally, the patient was educated on appropriate sun protection. The patient’s skin lesions subsided during the 2-month treatment. The oncologist switched the treatment from ribociclib to palbociclib, and no dermatological adverse effects were subsequently noted.

## 3 Discussion

As reported by Yang L et al., CDK4/6 inhibitors are associated with adverse events, such as neutropenia, leukopenia, thrombocytopenia, anemia, fatigue, diarrhea, febrile neutropenia, nausea, and elevated alanine aminotransferase levels (9). Ribociclib, a CDK4/6 inhibitor, also has a similar side effect profile, especially in relation to hematological problems. In addition, severe dermatological side effects, such as skin rash, vitiligo, and alopecia, have been reported in recent years (7, 10); however, these have been relatively less documented. To date, few cases of severe ribociclib-induced dermal toxicity, such as those of the Stevens–Johnson syndrome, toxic epidermal necrolysis, and erythema dyschromicum perstans-like pigmentation, have been reported in the literature (11–13).

Several cases of ribociclib-induced dyschromia, such as those of vitiligo (14–16), have been reported; however, the detailed mechanisms involved, including pathological examination and immunohistochemistry findings, are not well understood. Hence, we examined the results of Fontana–Masson staining and HMB-45 staining in the present case. Both staining methods revealed a reduced melanocyte number and the presence of melanin in the dermis. Besides, another report revealed the effect of ribociclib-induced pyknosis in keratinocytes (17); we also found dyskeratotic cells in the epidermis in our case. Therefore, we speculated that ribociclib-related dyschromia resulted from the uneven distribution of melanin and a decreased number of melanocytes, which are caused by impaired keratinocytes and melanocytes in the basal layer zone. The toxicity of ribociclib to these cells may originate from its inhibition of CDKs. However, further research is needed to validate this.

In the present case, the patient developed pigmented patches on the trunk and extremities, particularly over the forearms, 7 months after receiving ribociclib. Skin biopsy revealed interface dermatitis with melanin incontinence and clefting at the epidermal–dermal junction, similar to that observed in subepidermal bullous disease. The patient was diagnosed with phototoxicity with type IV hypersensitivity and subepidermal bullous dermatosis based on positive immunohistochemical staining for CD1a, CD3, CD4, and CD8.

To overcome the dermatological side effects of ribociclib, we first ceased ribociclib administration. To ensure continuation of anti-cancer therapy, we experimentally replaced ribociclib with palbociclib after obtaining the patient’s consent. Thereafter, we closely monitored the patient and educated them about sun protection; the dyschromia with bullae formation eventually subsided. The beneficial influence of this change may be attributed to immunotolerance or basic differences in the effects of CD4/6 inhibitors (such as subtle differences in the kinase selectivity between the two drugs); however, further studies are required to confirm this.

Drug-induced photosensitivity has attracted growing interest in recent years (18). Photosensitivity is categorized as either phototoxic or photoallergic. It occurs due to photosensitizing agents and subsequent exposure to ultraviolet or visible light. A previous study revealed that the photosensitive effect of CDK inhibitors was through the inhibition of ATP-binding cassette G2 (19). One case report also revealed ribociclib-induced photosensitive skin lesions in a patient with metastatic breast cancer (20). To our knowledge, the present case is the first on ribociclib-induced phototoxicity.

CDK inhibitors represent a novel class of targeted therapies for cancer treatment. However, they are associated with severe adverse effects that should be monitored for meticulously. In the present report, we revealed an association between ribociclib and phototoxicity with subepidermal bullous dermatosis and hyperpigmentation; this is a rare expression of drug allergy. Therefore, it is important for clinicians to closely monitor for cutaneous side effects and impart education on sun protection during clinical treatment with CDK4/6 inhibitors.

## Data availability statement

The original contributions presented in the study are included in the article/**Supplementary Material**. Further inquiries can be directed to the corresponding author.

## Ethics statement

The studies involving humans were approved by Hualien Tzu Chi Hospital, Buddhist Tzu Chi Medical Foundation Research Ethics Committee. The studies were conducted in accordance with the local legislation and institutional requirements. The participants provided their written informed consent to participate in this study. Written informed consent was obtained from the individual(s) for the publication of any potentially identifiable images or data included in this article. Written informed consent was obtained from the participant/patient(s) for the publication of this case report.

## Author contributions

J-YJ and W-EW wrote this manuscript. C-HCha diagnosed and treated the patient as well as reviewed and edited the manuscript. S-CC provided the case and clinical explanation. C-HChe assisted with pathological slide preparation and immunohistochemistry. All authors contributed to the manuscript and approved the submitted version.
