# Supplementary material for: Case Report: Ribociclib-induced phototoxicity presented as dyschromia with subsequent bullae formation
Source: Front Oncol. 2023 Aug 24;13:1184738. doi: 10.3389/fonc.2023.1184738 (PMC10484504; doi:10.3389/fonc.2023.1184738)
Supplement: Supplementary file 1 [file DataSheet_1.docx]

Supplementary Material


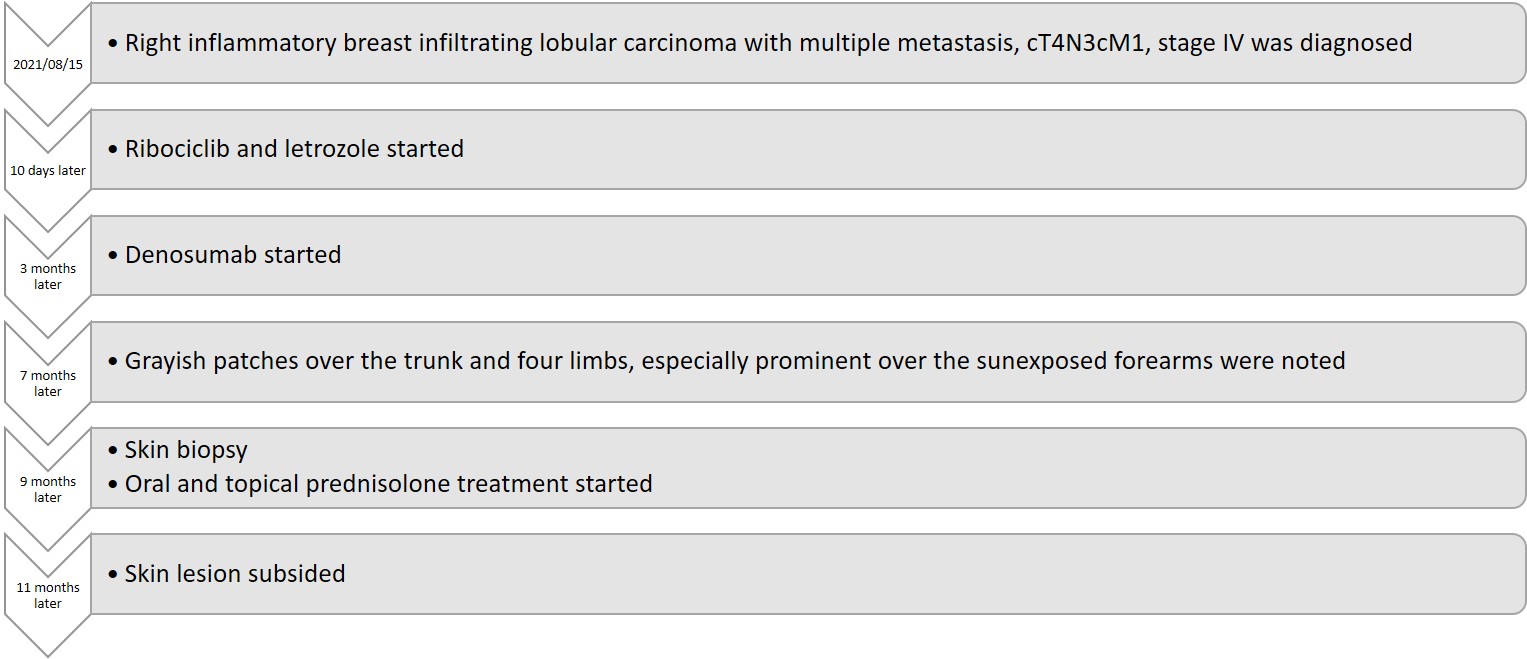


**Supplementary Figure 1**: Clinical course of the patient


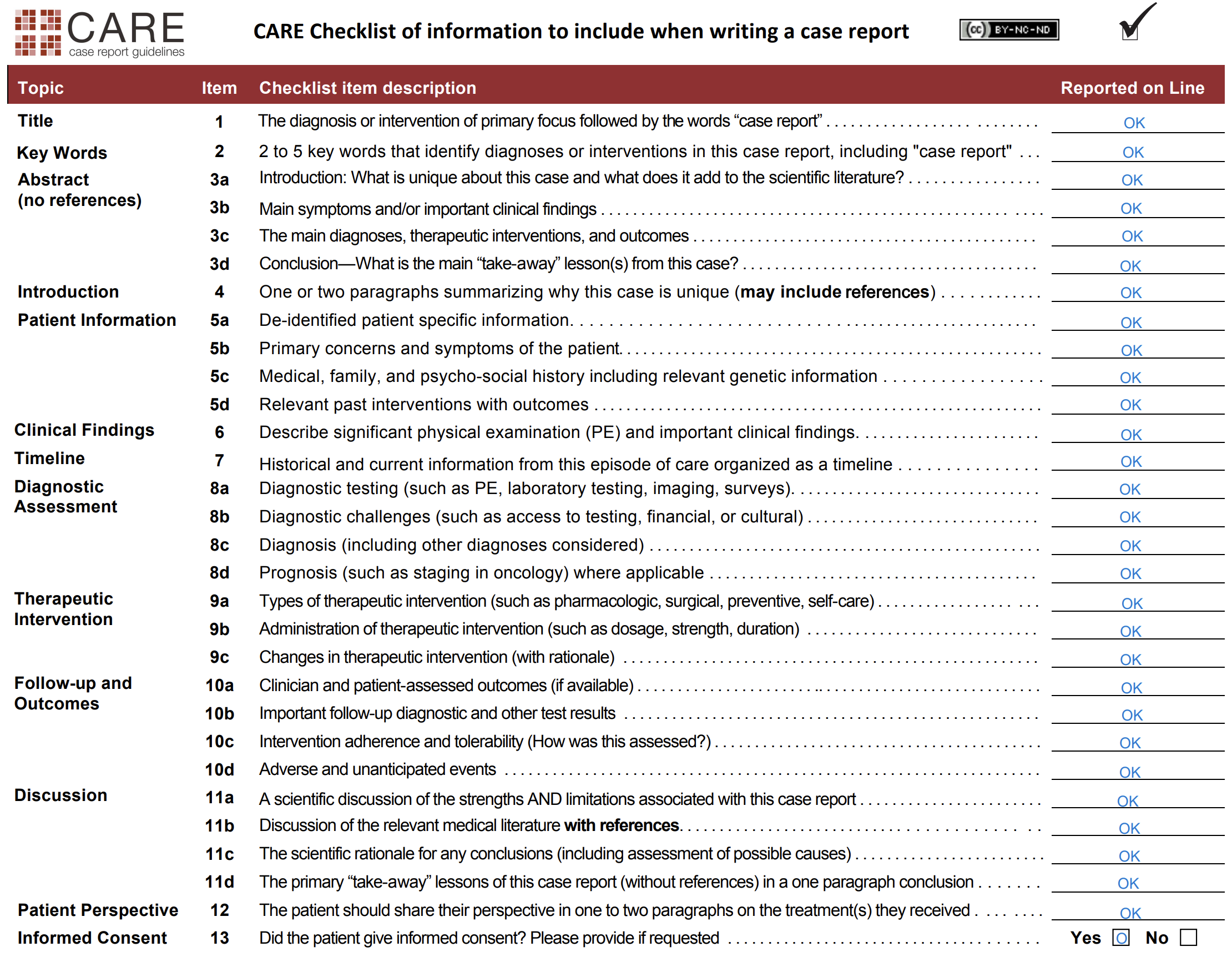


**Supplementary Figure 2**: Care checklist
